# “I Am Longing and Afraid to Depend on You”: A Case Report on Breakdowns of Therapeutic Alliance and Interpersonal Cycles in Complex Trauma

**DOI:** 10.3390/brainsci14121207

**Published:** 2024-11-28

**Authors:** Carolina Papa, Erica Pugliese, Claudia Perdighe, Ramona Fimiani, Francesco Mancini

**Affiliations:** 1 Associazione di Psicologia Cognitiva APC e Scuola di Psicoterapia Cognitiva SPC, 00185 Rome, Italy; e.pugliese@uva.nl (E.P.); perdighe@apc.it (C.P.); ramona.fimiani@uniroma3.it (R.F.); 2 Department of Psychology, Sapienza University of Rome, 00185 Rome, Italy; 3 Department of Clinical Psychology, University of Amsterdam, 1018 WS Amsterdam, The Netherlands; 4 Department of Education Science, University of Roma Tre, 00185 Rome, Italy; 5 Department of Human Sciences, Guglielmo Marconi University, 00193 Rome, Italy

**Keywords:** complex trauma, pathological affective dependence, fear of intimacy, therapeutic alliance, therapeutic relationship, interpersonal cycles, impasse, case report

## Abstract

Background: Patients with Complex Trauma (CT) may have an impaired ability to trust others and build intimate relationships due to non-integrated representations of self and others. This sometimes leads to an oscillation between needing and fearing intimacy in their adult relationships. This dynamic can occur in the therapeutic relationship, undermining the effectiveness of therapy and affecting the mental health of both the patient and the therapist. To date, no study has analyzed interpersonal patient–therapist dynamics in cases of CT. The present case aims to fill this gap by exploring relational cycles between the therapist and the patient during the therapeutic process in terms of goals and self–other beliefs. Methods: The methodology consisted of a shared and integrated reconstruction by the patient and therapist, both with clinical expertise in psychology, of the impasse in their therapeutic relationship. The reading was done through the lens of the cognitive model of Pathological Affective Dependence, a theory of traumatic relationships, by describing the primary interpersonal cycles occurring in the therapeutic relationship (altruistic, deontological, and vulnerable). Results: The condition of CT leads to several alliance breakdowns and specific interpersonal cycles, leading to new healing meanings for the patient and the relationship itself. Limitations: The study’s main limitation is that it consists of a qualitative analysis of the therapeutic relationship without data that can quantify the clinically observed changes. Conclusions: This case report demonstrates how CT, PAD and the fear of intimacy can be risk factors for the therapeutic alliance and how the therapeutic relationship constitutes a fundamental tool for intervention effectiveness in patients who experience unmet primary needs.

## 1. Relational Functioning in Complex Trauma and Fear of Intimacy

Building intimate relationships with others is one of the main characteristics of healthy personality development and one of the leading indicators of mental health [1]. Indeed, relationships are considered more satisfying when one feels intimate with another [2], and intimacy has been shown to play a protective role for biological, psychological, and social well-being [1,3]. Achieving a certain degree of intimacy involves sharing one’s vulnerability and assuming the potential risk involved. These actions will depend on the sense of trust built up early in meaningful relationships [4]. Intimacy has been described not only in terms of mutual self-disclosure, caring, warmth, and protection, but also in terms of relinquishing control, dropping defenses, and suffering at times of separation [5,6]. Reluctance to enter into intimacy consists of fear of exchanging meaningful personal thoughts and emotions with another individual who is of value to oneself and varies according to the importance attributed to the interaction partner [6]. More recently, Sobral and Costa [7] discussed that the fear of intimacy may not only be the fear of sharing specific personal thoughts or emotions but, also, the fear of dependence, which is experienced as fusion and loss of self. The ability to become intimate with others falls within the broader construct of dependence, a manifestation of attachment that aims to obtain care and support from an attachment figure within a secure relationship [8]. This ability is inadequately regulated in Complex Post-Traumatic Stress Disorder (c-PTSD). The ongoing micro-traumatic relational experiences, related to systematic distortions in early interactions between child and caregiver, influence how the person will interpret and react to interpersonal cues [9]. Indeed, c-PTSD refers to a condition resulting from repetitive and chronic traumatic events that impair personality development and fundamental trust in relationships [10]. Complex trauma has also been described as an impairment in an individual’s personal development that occurs during a critical developmental window in childhood, when self-definition and self-regulation are formed [11]. Some argue that complex trauma can occur even in adulthood from events such as war, war captivity, refugee status, human trafficking and prostitution, and acute or chronic illness [12]. Others stress that it results from an ongoing exposure to micro-traumatic events, such as emotional neglect, punishment, humiliations, or physical abuse. Indeed, traumatic events can encompass physical, sexual, and emotional abuse, as well as neglect and witnessing violence [13]. When events of cumulative interpersonal violence, neglect, and abuse occur during childhood by primary caregivers or attachment figures, children develop an insecure attachment style that will impact their adult relationships [14]. In line with this, psychological elements that characterize c-PTSD include exposure to chronic, prolonged, and repeated interpersonal trauma and abuse, attachment failure, inadequate sense of self, altered schemas, and emotional and impulse control dysregulation [15]. Insecure attachment leads individuals with complex trauma to have an impaired ability to trust others and build intimate relationships, which intensifies attachment anxiety and manifests as a fear of rejection, avoidance of relationships and fear of closeness [4]. Indeed, people with complex trauma may manifest the fear of intimacy (FoI), intended as the tendency of avoiding situations that stimulate attachment needs [16]. FoI is characterized by denial of one’s needs and desires, contempt for dependence on oneself and others, and hesitation to seek help to minimize attachment disruptions [17,18]. This construct can be considered the flip side of the condition of Pathological Affective Dependence (PAD), defined by Pugliese et al. [19,20] as a relational condition in which the main goal is maintaining relationships at all costs, even if it is a source of suffering for at least one of the people involved. According to PAD theory [19,20], the primary needs that people wish to first satisfy in the initial relationship, and then in the adult relationship, are threefold: the need for love, dignity, and safety. Conversely, the deprivation of these basic needs can lead to an idea of the other as violent, humiliating, and excessively fragile. PAD and FoI can be considered two symmetrical conditions in that, both underlie a difficulty in regulating dependency and care needs in relationships. Although both arise in complex trauma from the dissatisfaction of primary needs for love, dignity, and safety [19], they are also opposites and differ in coping mechanisms: one denies those needs, while the other over-invests in their satisfaction l. Complex trauma can manifest at the interpersonal level through extreme need of dependence as in fusional relationships or in the need of avoidance of closeness and intimacy, as it is perceived as too frightening [16].

## 2. Therapeutic Relationship in Complex Trauma and Fear of Intimacy

The unmet relational needs and the resulting image of the other can be extended to all intimate relationships, including therapeutic ones [21]. The therapeutic relationship represents a fundamental healing tool: the relational context that previously represented suffering, betrayal, and abandonment becomes an opportunity to modify the patient’s relational models and instill a sense of security through the presence of the therapist [22]. This may vary to the extent that the therapist provides a coherent, safe, and protective relationship. As such, FoI may be a risk factor in the patient’s and therapist’s ability to establish a good therapeutic alliance due to the patient’s tendency to avoid closeness with the therapist [23]. In these patients, the possibility of depending on others and entering into intimacy is perceived as dangerous because it coincides with the risk of being abused, mistreated, or abandoned again. Behind this fear lies a strong desire for dependence and care in relationships, as a consequence of their unsatisfied needs for a secure attachment. However, those people believe that they will be rejected if they reveal their true nature and deep needs, and they may be challenged about the possibility of being accepted and receiving love [24].

The activation of attachment needs in complex trauma leads the patient to criticize themselves, adopting a position of detachment or hostility and contempt towards the therapist, leading the latter to experience inadequacy, anger, and the instinct to reject the patient. For this reason, they are reluctant to self-disclose and may engage in strategies of positive self-presentation or self-silencing [25,26]; they may protect themselves from expected rejection by devaluing or withdrawing [27]. The patient may also become demanding, making unrealistic requests of the therapist that go beyond the therapeutic relationship itself. When the therapist confirms the patient’s negative image (e.g., showing their impatience or intolerance) or when they fail to meet the patient’s idealized expectations (e.g., by setting proper boundaries in the therapeutic relationship), the patient could experience a state of hopelessness and helpless anger that leads to disorganization on an emotional, cognitive, and behavioral level [28]. These representations, if adequately grasped, provide crucial information on the patient’s traumatic attachment experience. On the contrary, problematic interpersonal cycles occur in the therapeutic relationship if the therapist unintentionally confirms them. Interpersonal cycles produce intense processes of hyper-involvement, competition, rejection, or detachment in the relationship. We want to stress that the cycle activations often don’t depend on the experience or professional orientation of each therapist. Rather, it is more related to the fact that working with difficult patients is complex and can lead to therapist burnout. Addictionaly, the content of these cycles depends not only on the patient’s pathogenic interpersonal models but, also, on metacognitive aspects such as integration difficulties, which lead the cycles to be rapid, intense, changeable, and difficult to predict [29]. The difficulty of integration refers to the lack of a coherent and integrated system of one’s and others’ representations regardless of the variability of mental states [30]. Self–other representations can be particularly incongruous when a caregiver was both a source of and solution to alarm; thus, fear tends, paradoxically, to coexist with the calming action provided by proximity to the caregiver [31].

## 3. The Current Study

Despite the relevance of the therapeutic alliance for psychotherapeutic outcomes and the evidence that avoidance of intimacy is a risk factor for treatment effectiveness [32,33], few studies have thoroughly examined the relational dynamics that emerge in the therapeutic relationship when the patient struggles against the need to depend on the therapist, who represents both a source of desire and fear. The main innovativeness of the present contribution is that, for the first time (from what we know), patient and therapist, both with clinical expertise in the psychological field, collaborate in an integrated reading of their therapeutic relationship, characterized by continuous processes of impasse in and repair of the alliance as well as by an alternation of moments of intense emotional closeness and intimacy with others charged with destructive anger and the desire to repel each other. The therapeutic setting leads the patient to relive different aspects of her traumatic attachment, taking the form of a courtroom in which both the patient and the therapist remain for a long time trying to find an answer to the question: who is the victim; who is the abuser? [34]. The interpersonal cycles that occur in the therapeutic relationship are shown, as well as the elements that lead to the breaking of the alliance and the elements that, on the other hand, favor its repair and the process of integrating the chaotic representations of self and therapist into the patient’s mind. Specifically, the study’s aims are as follows:

1. Analyze interpersonal functioning in complex trauma through a clinical exemplification, with a specific focus on the therapeutic alliance;
2. Understand the impact that relational trauma has on the therapeutic alliance and, consequently, on the therapeutic process.

The therapist’s interventions aim to foster self-awareness and self-regulation through the therapeutic relationship and to move toward a more regulated dependency and more functional relational behaviors [35]. The therapist intervened using CBT, specifically with the following type of interventions; e.g., sharing the patient’s psychological functioning and cognitive restructuring. A detailed description of all interventions is not included as it is not the focus of this paper, which is aimed at highlighting breakdowns in the therapeutic alliance.

The interpersonal cycles that occur during the several breakdowns of the therapeutic alliance are described on the basis of the cognitive model of PAD [19] and the construct of Fear of Intimacy (FoI) [6]. We chose to base our analysis on PAD theory because it provides a strong framework to understand the relational difficulties in individuals with complex trauma. Both PAD and FoI explain how emotional dependency and fear of intimacy manifest in maladaptive relational patterns that complicate the therapeutic alliance. Patients with complex trauma often struggle with emotional vulnerability, leading to withdrawal or sabotaging behaviors that disrupt the treatment process. By integrating PAD and FoI, we can better understand the interpersonal cycles that emerge during therapeutic breakdowns and offer more effective understanding of those unexplored conditions.

This study is based on a qualitative analysis of a clinical case, focusing specifically on the direct observation of therapeutic interactions and the progression of the therapeutic process. The primary data were gathered through clinical observations made by the therapist during sessions and, subsequently, integrated with evaluations from an external supervisor (who was a psychotherapist specialized in c-PTSD). The supervisor’s role was to assess the coherence and integration of the information provided by both the patient and the therapist once the therapeutic treatment was concluded. Quantitative data were not collected, as the study relies primarily on the subjective and relational dynamics observed within the therapeutic setting. This approach aims to capture the complexity and nuance of the interpersonal processes inherent to unregulated dependency and therapeutic breakdowns. Lastly, the re-reading of the present case results from the collaborative empiricism operationalized between patient and therapist with the shared goal of reporting their story as a clinical exemplification of the therapeutic relationship in the context of complex trauma arising from early dysfunctional relationships. Additionally, joint review of therapeutic progress and the writing of this work, supported by a third-party supervision, improved collaboration and ensured coherence and the integration of insights. The patient has given written informed consent for publication.

## 4. Case Formulation

The case formulation is based on an integrative approach, combining PAD theory [19] and the FoI conceptualization [6]. The patient’s relational patterns and emotional regulation difficulties are understood through unresolved trauma and how these dynamics have shaped her fear of intimacy and her struggle with interdependence. Within this framework, the patient’s tendency to experience relationships as excessively dependent, controlling, and emotionally abusive is explored. These patterns stem from an underlying fear of intimacy, which causes her to avoid showing emotional vulnerability. This fear of being emotionally exposed leads her to interpret any attempt to reveal her true self as potentially dangerous, reinforcing a cycle of emotional avoidance and relational dysfunction.

The patient is a young woman and works as a psychologist in a psychiatric community. She decides to start therapy one year after her grandmother’s death, who died suddenly of a heart attack. The patient initially reports experiencing a significant difficulty in ending a relationship with her friend, which she recognized as dysfunctional and unhealthy for her but from which she cannot extricate herself because of the guilt she feels at the idea of doing so. She complains, therefore, of a lack of assertiveness that makes her feel compelled to stay in the relationship, generating anxiety and insomnia and often leading her to give up her needs and desires. The patient has central beliefs that guide her behavior; in particular, she has the idea that expressing her needs makes her a bad person and that this coincides with the other person’s unhappiness. From the perspective of a finalistic model of mind, the patient’s goals refer to motivations and plans that guide individual functioning [36]. In the field of psychopathology, in particular, the patient’s over-invested anti-goals assume relevance: these constitute scenarios and internal states experienced by the patient as unacceptable in their subjective representation [37]. The patient’s main anti-goal (the worst case scenario that she wants to avoid at all cost) initially emerges as generating suffering in the other in view of fulfilling her legitimate needs. She imagines that communicating her suffering to the friend will deeply hurt her and make her feel responsible for the patient’s suffering. The patient, according to the clinical observation of the therapist and an external supervisor experienced in complex trauma, falls within the ICD-11 criteria for c-PTSD. Specifically, during her childhood she was repeatedly exposed to traumatic events of an interpersonal nature and attachment failures. She exhibits emotional regulation difficulties and chaotic self/other representations [15]. The patient also appears to suffer from Pathological Affective Dependence (PAD) in her relationship with the friend, specifically reflecting the “Savior” profile [19]. PAD is characterized by a dynamic where at least one person in the relationship experiences significant suffering, yet they remain in the relationship for various reasons—fear of losing dignity, concerns about safety, or worry about causing suffering to a significant other. This last aspect seems to perfectly describe the patient’s experience.

The patient grew up with a completely blind brother in her family. The presence of disabled children can be a source of great stress within a family system and indirectly impacts the functioning of the parental couple, leading to chronic emotional and behavioral problems in the non-disabled sibling/s [38]. Many of these siblings feel that they do not receive the same amount of attention from their parents due to the demands of the other sibling’s condition and perceive that they receive unfair treatment and at a lower standard compared to the sibling [39]. They also often feel survivor guilt, which is the feeling of being spared the harm of others and the idea of having some kind of advantage over them, i.e., a better health [40]. These children are often expected to act as a parental figure for their sick sibling [41]. This is in line with the patient’s pathological belief that love can be expressed only through self-sacrifice with the aim of avoiding the other’s suffering [19]. Due to the chronic critical condition of the brother, the parents were mainly focused on him, neglecting the patient’s emotional needs of love and dignity [19]. This means that she was often asked to give up rights that her brother could not access. When she was allowed to enjoy those rights, she was instructed to hide them from him and remain silent to protect him from feeling hurt. This led the patient to develop the belief that the expression of her needs would only cause suffering to the others. For example, when the patient attempted to step out of her role as a savior by asserting her right to be seen by her mother, the mother became guilt-ridden, humiliated the patient, and threatened abandonment. Similarly, when the patient became avoidant and detached to dissociate from the negative emotions that her mother triggered, the mother responded with stressing and over-controlling behaviors. This reinforced her idea of a relationship as not attuned but, rather, based on constriction, driving the patient further to avoid intimacy. The sudden grandmother’s death triggered the patient’s pre-existing vulnerabilities, leading her to experience new trauma resulting from the loss: she couldn’t save her grandmother and she was suffering terribly. Despite the patient’s efforts, she believed she had failed in her most important goals: (1) saving others and preventing their suffering; (2) protecting her dignity, as she felt vulnerable and needy; (3) feeling safe, as she realized she had lost a major protective figure for her.

The patient’s vulnerabilities, the relationship goals she desires to achieve, and the fears she tries to avoid align closely with the constructs of PAD [19] and the Fear of Intimacy (FoI) [6]. This pattern reflects how the patient seeks connection and support while simultaneously struggling with deeply rooted fears of intimacy and emotional exposure, which are core components of both PAD and FoI. As a result, she oscillates between these two conditions, unable to regulate her need for dependence, leading to relational difficulties and emotional distress.

**Figure 1:**
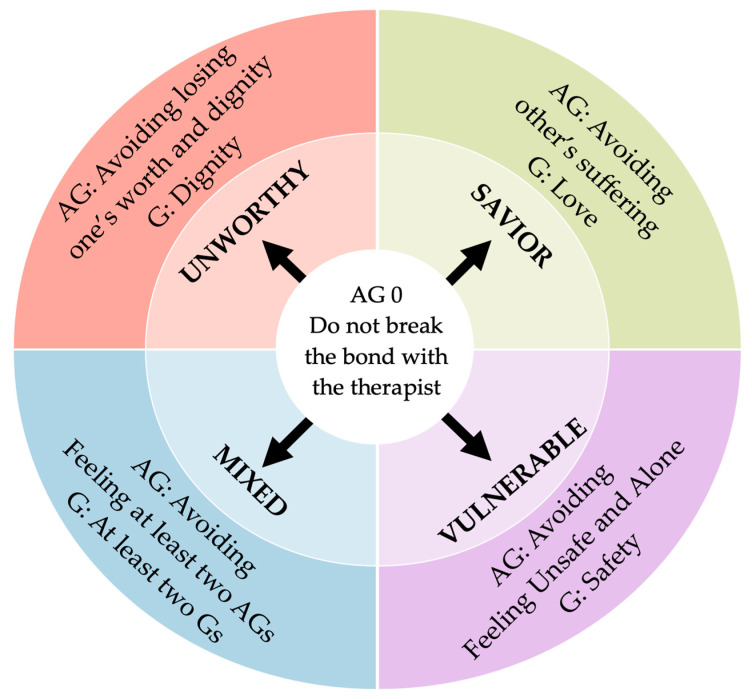
Cognitive model of Pathological Affective Dependence (PAD) applied to the therapeutic relationship [19,35]. Note: G = Goal; AG = Anti-Goal; Gs = Goals; AGs = Anti-Goals.

In light of the psychological functioning just described, the authors interpreted the patient’s mental functioning according to the cognitive model of Pathological Affective Dependence, a mental health condition frequent in adults with c-PTSD [19]. The formation of several non-integrated pathological parts are the result of the failure to meet basic needs for love, dignity, and safety during early relationships with problematic adults. According to the model, there are four types of parts that past adverse relationships can generate, frustrating the three basic relational needs (love, dignity, and safety). Each part is characterized by one of the three needs, mainly frustrated, during both early and current relationships. They are named Savior, Unworthy, Vulnerable, and Mixed (see Figure 1). Specifically, the Unworthy part arises from the frustration of a person’s need for dignity and feeling valued by significant others. This frustration can stem from a lack of recognition or validation, leading to feelings of unworthiness. When it is active, the person attempts to defend their dignity over another, which is perceived as humiliating. The Savior part is originated by the frustration of the need of being loved in favor of another who is (excessively) perceived to be more in need and fragile. When this part is active, the person tends to sacrifice themselves to prevent the other’s suffering. The Vulnerable part is marked by the frustration of the need to feel safe, healed, and protected by significant ones. When it is triggered by a perceived abusive other, the person actively tries to avoid loneliness and abandonment. The Mixed part shares at least two of the three main needs with frustrated and associated behavioral patterns. This last part activates the most complex interpersonal cycle [19]. In line with the above described condition of FoI, the patient is afraid to enter into intimacy with others. She shows an inhibited capacity to exchange meaningful personal thoughts and emotions with a significant other. The origin of this condition can be traced back to the repeated traumatic events experienced during childhood, specifically in the relationship with her mother. Both the fear of intimacy and the fear of losing others she experiences in most of her relationships, is constantly reproduced in that one with the therapist. The patient’s behavioral patterns, particularly her self-sacrificing tendencies and difficulty expressing her needs, are fully projected onto the therapeutic relationship. Just as her mother controlled and invalidated her needs, the patient may replicate these dynamics with the therapist, either by suppressing her own needs or feeling guilty for expressing them. This transference often plays out in the therapy setting, where the patient expects the therapist to become either neglectful or overly controlling, mirroring the relational patterns formed in her family. These projections hinder the therapeutic process, leading to several impasse between them over time. The patient’s expectations, shaped by past relational experiences, make it difficult for her to trust the therapist fully or feel safe in expressing her needs.

This happens precisely when the therapist informs the patient that she has to interrupt therapy for a few months because of her pregnancy. This event will generate the first major rupture in the therapeutic alliance and will lead to a long impasse in the therapeutic process. Indeed, this event constitutes a new traumatization for the patient: when the patient began therapy, she exhibited symptoms of PAD, often forming relationships with distant and problematic individuals, mirroring her caregiving role toward her blind brother. As the therapeutic relationship progressed and the therapist took on a caring role, the patient’s fear of intimacy was activated. This fear became particularly apparent during the therapist’s pregnancy, which, for the patient, symbolized the risk of losing her therapist, and being abandoned entirely. The patient’s fear of intimacy was heightened by the perceived threat of losing the therapist’s care, creating a significant obstacle to developing a healthy and balanced dependent relationship.

In line with PAD theory [19], we can expect that this event would activate several self–other images and interpersonal cycles [35]. For instance, when she feels a sense of loneliness and danger due to the therapist’s distancing, she imagines the therapist as not caring of her needs (Vulnerable Self/Abusing Other). She also feels unable to express her suffering for fear of damaging the therapist (Altruistic Self/Fragile Other). At the same time, she fears confronting her own suffering, as doing so could make her feel dependent on the therapist and vulnerable to the same devaluing reactions she has always received from her caregiver when expressing her needs (Deontological Self/Humiliating Other). The patient continually brings these beliefs and fears into the therapist relationship, hoping she can take care of them. The patients may refuse pathogenic beliefs; i.e., “*My needs are a burden on others*”, developed during childhood, to achieve healthy and adaptive goals [42]. One of the main ways the patients use to disprove their pathogenic beliefs is to test them in the therapeutic relationship through relational tests [43]. The patient, unable to express her feelings directly, tests the therapist to see if they are ready to accept her emotions. To pass this test, the therapist must respond in a way that disproves the patient’s negative belief [44]. If the therapist fails the test, it can trigger cycles in the relationship that reinforce the patient’s emotional pain [29].

The aim of this study is to describe these moments by identifying typical interpersonal cycles in the therapeutic relationship with complex trauma.

## 5. Interpersonal Cycles

Some of the best-known models have conceptualized the therapeutic alliance as the place where the patient re-proposes their relational patterns in the relationship with the therapist [45] in order to disconfirm their pathogenic beliefs [42,43]. Ruptures in that relationship are enactments shaped by dissociated aspects of the patient’s and therapist’s experiences in mutual interaction [46]. Pugliese et al. reconstructed the interpersonal cycles that occurred in the therapeutic relationship by framing the patient’s functioning within the cognitive model of Pathological Affective Dependencies [19]. Indeed, the double bind inherent in maltreatment by a caregiver leads to the development of alternating and dissociated self-states with contradictory attachment models, such as idealizing/devaluing or victim/persecutor. The therapist can serve as a relational bridge between these states, allowing the patient to internalize an integrated model of the therapeutic relationship [47]. Bearing in mind that the patient may react to their dysfunctional belief by resigning or rebelling against it, as well as by making the other person feel the way they feel, the following describes the interpersonal cycles that typically occur in the therapeutic relationship between patient and therapist (for a detailed analysis of the interpersonal cycle according to PAD theory, see Pugliese, 2024 [35]). The cycles described below are not to be considered as occurring in a temporally sequential manner but, rather, in a chaotic and alternating manner. Indeed, the activation of the three traumatic parts leads to the activation of a complex, mixed cycle that is self-feeding over time, characterized by moments in which the patient’s sense of devaluation is followed by that of the therapist, that of harming the therapist follows the fear of hurting the patient, and the patient’s sense of danger is followed by that of the therapist. The unity of mental states and the complementarity of the roles that the patient and therapist embody are characterized by a continuous alternation of moments of mismatch and repair in their relationship, leading to a prolonged impasse in the therapeutic process.

### 5.1. Deontological Cycle

**Figure 2:**
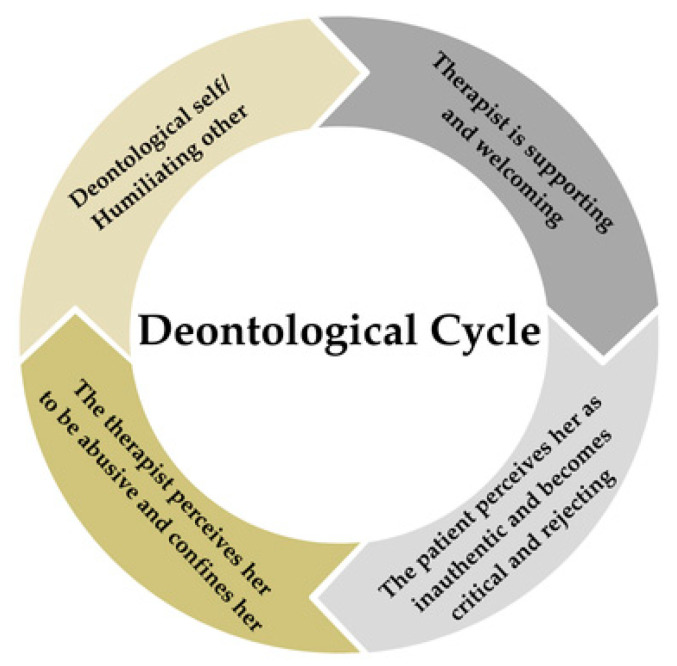
Graphic representation of the deontological cycle.

When the deontological part is active, the patient feels unworthy and worthless [19]. The belief that the therapist humiliates and despises the patient emerges whenever the patient comes into contact with her own attachment needs that are elicited by the therapeutic relationship. Not knowing any other way to express these needs, given that she has never been able to express them in the relationship with her caregiver, the patient uses the coping mechanism she is most familiar with; i.e., avoiding anything that could potentially stimulate the emergence of needs for affection and care. The continuous exposure to the closeness with the therapist, however, causes her usual strategy to collapse, bringing out all the dissociated emotions related to the patient’s history of emotional deprivation. Consequently, driven by distrust in the relationship, she repeatedly tests the belief that she is unworthy and does not deserve that closeness in the therapeutic relationship. The deontological cycle takes many forms in the case at hand, passing through moments when the patient surrenders to her schema [45] by showing the therapist her most undeserving side; e.g., that she drinks out of proportion, and is expulsive, and cares little for those around her, thereby eliciting the therapist’s discomfort in the hope that the therapist will convey the message that she deserves love, care, and protection [43]. Faced with the threat of loss of the relationship, triggered by separation from the therapist, the belief of unworthiness is challenged in the therapeutic relationship through motions of self-punitive overcompensation in which the patient rebels against her conviction by emphasizing her clinical skills in opposition to those of the therapist. The self-punitive component of this pattern derives from the fact that it tends to elicit rejection in the other, even though it implies a desire for protection. The therapist’s reactions are initially warm and welcoming. Still, the patient’s state of helplessness resulting from the traumatic experiences with the caregivers leads her to interpret them in light of her state of mind; that is, as fake, inauthentic, and not very credible. The more the therapist shows empathy and recognition towards the patient’s suffering, the more the patient accuses her of being fake and strategic: “*You are only acting this way because it is the right therapeutic strategy to follow, but you are fake because you hate me*”. The safety and attachment needs stemming from the traumatic bond with her parents leads the patient to dominate the environment through solution attempts to control all the therapist’s movements [28], arriving at a role reversal in which she tries to make the therapist feel unworthy, criticizing and devaluing her in her therapeutic work. The therapist initially tries to remain calm and suppress the reaction that the patient usually elicits from her. Still, the patient starts becoming provocative in the face of the therapist’s reception, as if making the therapist break down has become her sole purpose: “*Oh, thank you, sure, I can imagine how nice it must be to hear these things from a patient. Your gratitude seems believable... thanks for constantly doubting me week after week. It feels like such a joke. Just admit that you can’t stand me anymore and be done with it. Be coherent! Tell me to go to hell because we all know that’s what you want to do, and you’re only refraining from doing it because you can’t. Just do it, and let’s end this charade*”. The feelings of fear and distress that are activated in the therapist in reaction to the patient’s attitudes hinder her from taking actions aimed at alliance repair, such as inner-discipline, validation, and metacommunication [46]. Even when the therapist tunes in, the patient challenges her, insinuating that she sought supervision and her interest was not genuine. The therapist perceives the patient’s expulsive behavior as humiliating and feels the same unworthiness as the patient, indicating that she does not deserve to be in the position she is in, identifying herself with the victim; therefore, the therapist tries to defend herself and her therapeutic choices. She justifies to the patient the rationale behind her therapeutic approach, pointing out what the patient’s behavior elicits in others while simultaneously struggling, unsuccessfully, to conceal her annoyance. The therapist’s reactions confirm the patient’s hypothesis; the latter is focused on finding evidence to prove the pattern that the other will never be genuinely interested in her but, instead, find her disdainful. The therapist thus confirms to the patient the belief that, if she expresses her suffering, she will be despised; therefore, the patient has won. See Figure 2 for graphical representation of the deontological cycle.

### 5.2. Altruistic Cycle

**Figure 3:**
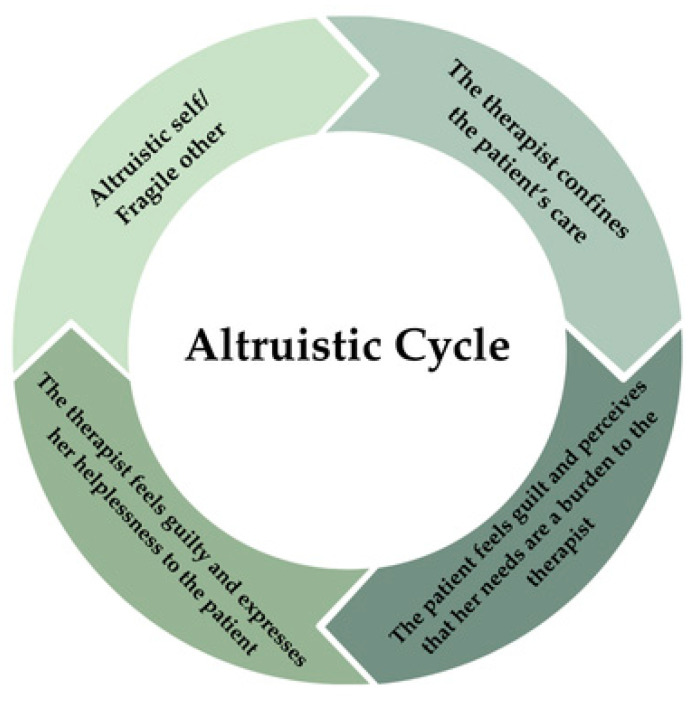
Graphic representation of the altruistic cycle.

The patient believes that her lovability depends on how much she sacrifices herself for the other, who is seen as fragile and needy [19]. In her mind, the expression of a need is a burden for anyone; thus, with the active goal of not making the therapist suffer, the patient tries hard not to burden her. To do so, the patient either does not express her suffering during the sessions or she belittles it with the idea that the therapist will already follow several patients who are more demanding and problematic than she is in order that the therapist will feel burdened. The therapist considers the reasons why the patient behaves this way, based on the family history reconstructed together, then tries to stimulate an opening by confronting the patient with her vulnerability, but the patient continues to avoid it. When the sudden separation due to the therapist’s pregnancy occurs, the patient would like to ask her for help but is unable to do so because of her altruistic schema. Confirming her schema, the patient has thoughts about the therapist’s fragile condition due to her pregnant state; e.g., “*she will have so many things to organize, she might be physically ill, she just misses me making demands on her at this time!*”. When the altruistic part is activated, the continuous monitoring of the therapist’s state (arising from the fear of losing her) leads the patient to explaining every failure of the therapist; e.g., if she seems distracted or in trouble, in light of her fragility during the pregnancy period, increasing the patient’s withdrawal. For example, when the patient knows that the therapist has essential things to do or celebrate, she might ask to change the appointment because she thinks the therapist deserves more time free from the patient’s burdensome demands that might ruin the time for her. When feeling sad or angry with the therapist about the separation, the patient would force herself to go against her feelings for fear of burdening the therapist or to protect her from possible negative emotions: “*If I were to disappear now, she would start questioning herself and wondering if she did something wrong, I would give her another thought beyond those she already has*”. Even when the therapist invites her to express her anger during the session by legitimizing that state and showing readiness to accept it, the patient withdraws and refuses, as she thinks, “*I could never hurt her so much*”. The non-expression of suffering and the request for help leads the therapist to fail to realize the emotional significance the separation has for the patient, confirming her idea of unavailability of care; i.e., “n*o one notices me if I am suffering*”. The patient then begins to experience therapy as a rehearsal in which she must succeed in suppressing her pain, session after session, in order to not contaminate the therapist with demands for help. The pattern repeats itself, leading the patient to act in the protection of the therapist and to experience her suffering alone once the session is over. The altruistic cycle is reactivated more forcefully later, when the patient rebels against her pattern by becoming excessively demanding towards the therapist, perceived to be unavailable, and trying to make the therapist feel the way she feels. The patient, therefore, accuses the therapist of not being attentive to the patient’s needs and wants, identifying with the victim. In light of her own altruistic goal, the therapist is unable to accept the patient’s criticism without blaming herself for something for which she is not responsible, identifying with the abuser. It is for this reason that the therapist, feeling helpless and guilty for not being able to give the patient what she needs, suggests that she interrupt the therapeutic relationship and turn to another therapist. Even if the therapist’s motivation was not to harm the patient but, rather, to offer a better option, this event constitutes a new traumatization for the patient, who confirms the idea that expressing her needs is a burden for the other and that doing so will lead her to be removed. The patient rebels again and does not allow the therapist to leave her and to make her relive what she has already experienced when she was a child, blaming the therapist and forcing the therapist not to abandon her, even though, by now, in her mind, she thinks the therapist is looking forward to it. See Figure 3 for graphical representation of the altruistic cycle.

### 5.3. Vulnerable Cycle

**Figure 4:**
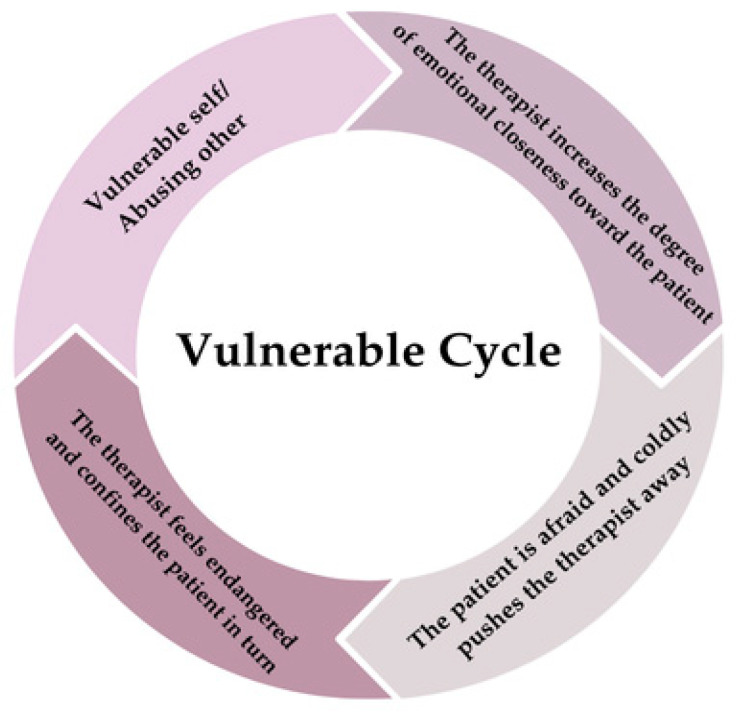
Graphic representation of the vulnerable cycle.

The patient vigorously tries to counter the belief about her vulnerability and fear of being abandoned in each relationship, including the therapeutic one. The more involved in the relationship she becomes, the more she is terrified to lose the bond with the therapist. This feeling is unacceptable for her and is often refused. This consideration activated the following cycle. When the patient feels endangered and unsafe, she imagines that the other person will not be willing to accept her and may even get angry. Indeed, the patient’s main goal is to avoid entering into intimacy for fear of showing her vulnerable part that would make her, in the representation of her suffering, dependent on the other and, thus, at risk of reliving emotional abandonment. Not only does the patient fail to express her emotional needs in her intimate relationships: she also tends to devalue, criticize, and feel repulsed by any aspect of healthy emotional dependency in herself and others. As stated above, emotional closeness with the therapist represents a trigger for the patient, who then avoids getting in touch with her attachment needs in order to achieve her goal. To do this, the patient avoids sharing aspects of personal fragility, changes the subject when the therapist broaches them, or openly refuses to talk about specific topics and feelings that are too emotionally activating for her, such as the event of her grandmother’s death. Even when the therapist tries another route by exposing the patient to imaginative techniques to recall painful memories, the patient tends to discontinue the procedure to the point of outright refusing to undergo it. The inability to express one’s vulnerability leads the body to become the sole vehicle for the intensity of the emotional experience with the therapist; therefore, the patient alters all her neurovegetative functions whenever she feels the therapist’s absence but cannot express it; e.g., she cannot eat or sleep, she presents constipation, and she gets stress rushes, etc. In this perpetual and painful struggle with the risk of being emotionally dependent on the therapist, the event of separation activates the patient’s sense of vulnerability and the feeling of concrete and real abandonment in her mind. The patient, therefore, continually lets other pathological parts emerge, sometimes identifying with the victim and sometimes with the abuser, in which she feels more in control. In this interplay, the therapist takes on roles similar and complementary to the patient’s, causing the patient to feel even more endangered at the idea of expressing her vulnerable part; e.g., imagining that the therapist gets angry or gives her a burden she will not be able to handle. The only way the patient finds to test her fear of being abandoned by the therapist is to once again reverse roles by destroying the therapist, despite the terror resulting from the consequent loss, and continuously seeking evidence in favor of her thesis; e.g., “*she is too fragile, she cannot handle a patient like me!*” At the same time, the patient cannot help but monitor the therapist’s movements. In the patient’s mind, the relationship can only exist through exclusivity in an all-or-nothing thought pattern, where “*either you are the only one for me, or you are invisible*”, because this is what she has learned in her life history. She, therefore, feels the need to constantly know where the therapist is, to anticipate potential abandonment, and to know who the therapist is with in order to ward off a scenario where she again loses the chance for an exclusive relationship. For example, even if she destroys the therapist’s image, if she sees the therapist online, she gets angry and has thoughts like “*Who the hell is she talking to?*”. This pattern is highly indicative of the disintegration stemming from the state of helplessness induced by early dysfunctional relationships. The patient expresses to the therapist her sense of dissatisfaction with the therapeutic relationship, which stems from the defensive, self-imposed limit of not being able to show her vulnerability and from feeling trapped in having to assume one of the uncomfortable roles she has always played in her life history, even in the therapeutic relationship. The fear of revealing her pain and the sense of helplessness in the face of any possible response from the therapist, which never seems sufficient to reassure her, leads the patient to continually withdraw from the relationship, threatening the therapist with the same abandonment the therapist has made her feel. The therapist perceives the patient as abusive and feels in danger at the idea of the patient violently dropping out of the therapy and leaving her alone in the relationship. She then wonders if she is making the right therapeutic choice. She envisages catastrophic scenarios about how the therapy can proceed and feels vulnerable and in danger, fearing that any choice she makes would be the wrong one. As is the case in every interpersonal cycle, the emotional states of the patient and the therapist mirror each other. The therapist, as in front of a mirror, thinks that she feels exactly as her patient in front of her. At that point, she decides that the only possible way to stimulate the functional part of the patient is to show her feelings and vulnerabilities at that moment. In doing so, the therapist, invested by the patient with idealized expectations, stops being the only possible reparative container for the patient’s pain. At that point, the patient, as in front of a mirror, can see her dysfunctional thoughts and how she is projecting these onto the therapist. For the first time, the therapist is only a human being in front of her and no longer just a means through which she can and should make compensation for the damage she has suffered in her childhood. See Figure 4 for graphical representation of the vulnerable cycle.

## 6. Discussion

In this case report, the relational dynamics emerging within the therapeutic relationship represent an exemplification of the effects of complex trauma, PAD and subsequent fear of intimacy on interpersonal functioning. Furthermore, the shared reconstruction between patient and therapist of what their minds thinking at each impasse moment allows, for the first time, a clearer understanding of the various components that adverse relational experiences can generate, starting from the unmet basic needs of love, dignity, and safety [19] and the non-integration of self and other representations. Difficulty integrating multiple and changing representations of self and others leads these patients to oscillate chaotically between extreme mental states. The therapeutic setting takes on the appearance of a courtroom within the patient’s mind, where she sometimes assumes the role of a stern and faultless judge and, at other times, that of a victim unjustly accused, having to fight to defend herself. Although traumatic memories are reactivated in the face of real elements, they are not exact replicas of what has happened in the patient’s life. They may include the patient’s fantasies and misperceptions at the time, excluding parts of the experience [48]. Guided by the interpretive processes that serve her in coherently resolving the state of uncertainty generated by the therapist’s absence [49], the patient exhibits typical behaviors and communications that elicit predictable responses [46]. It could happen that the patient hosts the therapist in that courtroom, unaware that she is assigning the therapist the role that confirms her dysfunctional belief and the therapist, unaware, embodies it perfectly, intertwining her fears with the patient’s. Their thoughts, feelings, and behavioral tendencies mirror each other, and may result in chaos, confusion, and suffering for both of them. Indeed, in interpersonal cycles, both participants feel disappointed and hurt: the negative interaction and pain that results from it are likely to constitute obstacles to metacognitive functioning that could help to break the cycle and get out of the impasse [50]. Despite the patient struggling against the possibility of emotionally depending on the therapist, she experiences deep needs for dependency and attachment in the therapeutic relationship for the first time, which are felt as intolerable and relentless. When memories related to traumatic experiences are evoked through separation from the therapist, the patient feels a sense of threat; the fear of staying in the relationship can take the form of an exaggerated perception of being abandoned, blamed, considered repulsive, and therefore ‘contaminating’ the therapist [16]. The patient experiences unsustainable feelings of fear at the slightest loss of coherence from the therapist, such as a change in an appointment or a new haircut and, like the child facing the sudden change of a mother’s expression, she struggles to make sense of that experience [51]. Throughout the treatment, a significant focus was placed on interventions such as chair-based techniques, empathic attunement, and reflective dialogues to explore and reprocess the patient’s emotional states. These methods aimed to facilitate an experiential understanding of the patient’s fragmented parts and to foster a deeper emotional connection. The therapist’s calibrated self-disclosure and the following collaborative empiricism both played a crucial role in creating an atmosphere of trust, modeling vulnerability, and reinforcing the patient’s capacity for emotional expression [52,53]. The use of chair-based techniques allowed the patient to externalize conflicting inner-voices and to engage in dialogues that promoted self-compassion and integration.

Specifically, the impasse resolution occurs mainly when the vulnerable part of the patient is shown after the therapist’s action of self-revelation. In this way, the therapist, who has realized that her activation is containable as the patient elicits it, helps the latter to recover metacognition through modeling [54]. The patient not only gains awareness of her projections onto the therapist by seeing their effect on her, but she also learns to communicate her fragility differently and more functionally. Moreover, the fact that the therapist first showed her fragility allowed the patient to gain a sense of security in the relationship as the therapist was giving her something very intimate and was not afraid of it; on the contrary, in doing so, she was showing the patient that she considered her to be someone of value to her. This time the match did not create interruption and destruction but a new connection in the fragmented nature of the patient’s wounded parts because defenses fall and there is no longer any coping strategy in place to protect oneself. Such emotional connection with another who is responsive and attuned to her suffering becomes such a strong and intense corrective emotional experience [55] that it paves the way for a new construction of meaning in which there is finally hope, and it is possible to slowly rebuild what has been violently interrupted. In some ways, the different parts of PAD theory, as also explained in the Internal Family System Theory [56], become the strategies put in place by the patient to survive and to protect the ‘exiled’ parts related to attachment needs, which are more susceptible to pain [57].

Given these patients’ deep-rooted distrust, alliance repair is an ongoing process throughout treatment. In a sense, the moments of disconnection and connection between patient and therapist mirror the attachment development process between parent and child, in which one transitions from attunement to interruption and repair. This process is fundamental in psychotherapeutic treatment with those who have experienced prolonged and repeated experiences of fractures. This is especially true for those who can’t even imagine the possibility of repair. This allows individuals to build a new belief that the relationship can be interrupted and restored, deeply modifying their relational schema [11]. In this case, the therapist’s holding transmitted through mirroring allows for the slow construction of intimacy with the primary object and integration of the self [34,58]. In light of this new interconnection, the patient can experience intimacy with another significant individual for the first time without the belief of “*falling apart, going crazy, and being unable to do anything about it*” [16] but, also, without denying and nullifying the need to be cared for [18]. This allows her to step towards building a healthy interdependence in her relationships, which have always been experienced as frightening, including, initially, the one with the therapist. Indeed, the experience of the long impasse and repeated interpersonal cycles over time led both patient and therapist to read each other’s behavior no longer in light of their vulnerabilities, transforming dysregulation into co-regulation over time. The relationship between patient and therapist becomes the primary working tool and the main aim of the intervention, taking on a highly therapeutic value as it becomes the only means for the patient to learn to construct new meanings.

## 7. Conclusions

The description of the interpersonal cycles occurring in the present case highlights how learning to regulate one’s activations within the therapeutic relationship constitutes a fundamental prerequisite for therapists working with patients with complex trauma, PAD and a high fear of intimacy but, also, it underscores the relevance of the quality of the therapeutic relationship with these patients. Furthermore, this is the first time fear of intimacy (FoI), i.e., fear of revealing oneself authentically and being dependent on the other [6,7] has been framed through a cognitive model, demonstrating how the goals and beliefs that guide functioning overlap with Pathological Affective Dependence (PAD), i.e., a condition in which one feels indispensably attached to an abusive partner [19,20,35]. However, the two conditions differ in coping instruments to achieve the same basic primary needs. Although this clinical case presents a novelty in analyzing the impasse of the therapeutic relationship in complex trauma, specifically in the interpersonal cycles between patient and therapist, it has limitations. As it is a single case, the dynamics in the relationship between patient and therapist could not be extended to every therapeutic relationship with patients with complex trauma, PAD and FoI. Furthermore, no tools were used to assess variables related to the therapeutic relationship before and after the impasse that would have helped better understand the mechanisms of change; e.g., working alliance and real relationship. Changes in the therapeutic relationship and the patient’s psychological functioning were detected only through clinical observation, which is a limitation concerning the reproducibility of the results. Nevertheless, the integrated cognitive reading between patient and therapist of their beliefs, emotions and behaviors allowed for a careful examination of their interpersonal cycles. These can occur in patients who present an intense FoI in general, providing an entirely new contribution to the analysis of impasse in the therapeutic process in these specific clinical conditions. Future studies in this area are needed with a focus on the relational factors of both patient and therapist that may contribute to impasse and drop-out for therapeutic efficacy but, also, factors that are protective. Furthermore, this clinical case highlights how interpersonal functioning can be differentially impaired based on specific peculiar characteristics that differentiate FoI condition from that of PAD, revealing the need to broaden the field of research in this area. Since the ability to build intimate relationships with others is a protective factor for biological, psychological, and social well-being in general [1,3], future research should investigate individual and interpersonal factors that hinder this natural process. The present clinical case proves the therapeutic relationship’s relevance for its effectiveness in improving the regulation of the need for dependence and autonomy in relationships. The collaborative empiricism operationalized between patient and therapist, engaged in the common goal of sharing their story to contribute to research in this field, constitutes the most significant testimony.

## Data Availability

The data are not publicly available due to privacy or ethical restrictions.
